# A novel and facile approach to obtain NiO nanowire-in-nanotube structured nanofibers with enhanced photocatalysis

**DOI:** 10.1039/c8ra01211c

**Published:** 2018-03-20

**Authors:** Yue Wang, Dan Li, Qianli Ma, Jiao Tian, Yan Song, Xue Xi, Xiangting Dong, Wensheng Yu, Jinxian Wang, Guixia Liu

**Affiliations:** Key Laboratory of Applied Chemistry and Nanotechnology at Universities of Jilin Province, Changchun University of Science and Technology Changchun 130022 China dongxiangting888@163.com +86-0431-85383815 +86-0431-85582574

## Abstract

NiO nanowire-in-nanotube structured nanofibers were easily and directly fabricated *via* one-pot uniaxial electrospinning followed by calcination process for the first time. Firstly, Ni(CH_3_COO)_2_/PVP composite nanofibers were prepared by a conventional electrospinning method, and then NiO nanowire-in-nanotube structured nanofibers were successfully synthesized by two-stage calcination procedure of Ni(CH_3_COO)_2_/PVP composite nanofibers which was determined to be the key process for preparing NiO nanowire-in-nanotube structured nanofibers. The NiO nanowire-in-nanotube structured nanofibers have pure cubic phase structure with space group of *Fm3̄m*, and the outer diameter and wall thickness of nanotubes and nanowire diameter are 130 ± 0.99 nm, 30 nm and 40 nm, respectively. Preliminarily, it is satisfactorily found that NiO nanowire-in-nanotube structured nanofibers used as photocatalyst for water splitting exhibit higher H_2_ evolution rate of 622 μmol h^−1^ than counterpart NiO hollow nanofibers of 472 μmol h^−1^ owing to its novel nanostructure. The possible formation mechanism of NiO nanowire-in-nanotube structured nanofibers is proposed. To evaluate the universality of this novel preparative technique, taking Co_3_O_4_ as an example, it is found that Co_3_O_4_ nanowire-in-nanotube structured nanofibers are also successfully fabricated *via* this novel method. The special nanowire-in-nanotube structure of the one-dimensional nanomaterials makes them have promising applications in catalysis, lithium-ion battery, drug delivery, *etc*. This manufacturing strategy has some advantages over other methods to form nanowire-in-nanotube structured nanofibers, such as easy, highly efficient and cost effective. The design idea and synthetic technique provide a novel perspective to create other nanowire-in-nanotube structured nanomaterials.

## Abstract 2

NiO nanowire-in-nanotube structured nanofibers were easily and directly fabricated *via* one-pot uniaxial electrospinning followed by designed calcination procedures.

## Graphical abstract

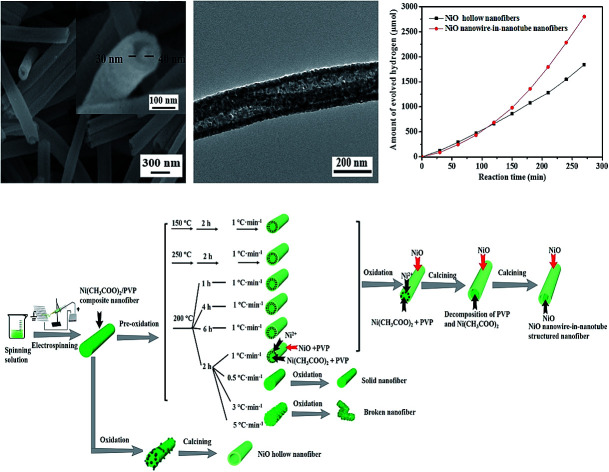

## Introduction

One-dimensional (1D) nanostructured materials have received increasing attention owing to their special properties, which have wide applications in various fields, such as magnetism, optics, photocatalysis, Li-ion battery, *etc*^1^. Many methods, such as hydrothermal method, electrochemical etching/deposition, solid state reaction, microwave synthesis, electrospinning,^2–6^ have been used to prepare these 1D nanostructured materials. Among these methods, electrospinning is a straightforward and effective method to prepare 1D nanomaterials with diameters ranging from micrometer to nanometer,^7^ including nanofibers/nanobelts, hollow nanofibers, coaxial nanofibers/nanobelts, Janus nanofibers/nanobelts, nanowire-in-microtube structured core/shell fibers.^8–16^

Recently, the nanocomposites with a hollow cavity between the shell and core have aroused widespread interest due to their special structure and large specific surface area, which have potential applications in biological medicine, sensor, lithium-ion batteries, adsorption and catalysis, *etc*^17–19^. Usually, the preparation process of these specially structured 1D nanocomposites contains three steps. The first step is the synthesis of core materials, and then the other two layers including intermediate layers and shells are gradually coated on the core materials to form triple-layered composite materials. Finally, the intermediate layers are removed by calcination or extraction using appropriate solvent, and the nanocomposites with void structure between the inner and outer substances are obtained. Tingting Wang *et al.* have prepared uniform yolk–shell architectures by high-temperature calcination of core/shell/shell structured nanomaterials to remove the middle layers.^20^ Nian Liu *et al.* have fabricated yolk–shell Si@void@C structure by using HF to remove SiO_2_ sacrificial layer in the Si@SiO_2_@C structure.^21^ In addition to the above three-step method, Zhao Yong *et al.* have reported the fabrication of nanowire-in-microtube structured core/shell fibers by multifluidic coaxial electrospinning approach. Firstly, three coaxial capillaries were assembled as the spinneret, and a chemically inert middle fluid was introduced to work as a spacer between the outer and inner fluids, and then a three-layered core/shell structure was formed. Subsequently, the middle layer of the as-prepared fibers was selectively removed, thus nanowire-in-microtube structured core/shell fibers were obtained with a hollow cavity between the shell and the core materials.^16^ However, the aforementioned preparation methods for nanocomposites with hollow cavity are mostly complicated and costly, and usually, the structural uniformity of the products is not satisfactory. Therefore, it is urgent to find a simple and efficient method to form nanocomposites with void structure in order to simplify the productive process and reduce costs.

NiO is p-typed semiconductor with wide band gap (3.6–4.0 eV),^22^ which has been widely used in catalysis, battery cathode, electrochromic films, *etc.* Mengzhu Liu *et al.* reported the formation of multilayer NiO nanostructures by electrospinning and compared the properties of multilayer NiO and NiO powders. The result shows that multilayer NiO exhibits much higher sensing signal than NiO powders due to its higher surface area.^23^ Xiong Wang *et al.* reported the formation, improved photocatalytic properties and excellent electrochemical performance of hierarchically structured NiO macroporous microspheres with large surface area.^24^ Hence, NiO with special structure possesses many excellent performances, which has been reported in the above literatures, especially it has been confirmed that NiO nanotubes have higher photocatalytic property than ordinary NiO solid nanofibers because of the larger specific surface area.^25^ Nanowire-in-nanotube structured nanofibers theoretically have larger specific surface area than counterpart NiO nanotubes. For this reason, the fabrication of NiO nanowire-in-nanotube structured nanofiber, as a novel and special morphology, is an important and essential subject to research. By now, no reports on the synthesis of NiO nanowire-in-nanotube structured nanofiber are found in the references.

In this work, we design a novel and simple strategy to form NiO nanowire-in-nanotube structured nanofibers by two-stage calcination procedure of electrospinning-made Ni(CH_3_COO)_2_/PVP composite nanofibers. The products were characterized systematically, and their photocatalytic water splitting activity was initially investigated, and some meaningful results were achieved.

## Experimental sections

### Chemical reagents

Nickel acetate tetrahydrate [Ni(CH_3_COO)_2_·4H_2_O, AR], polyvinyl pyrrolidone (PVP, K90, *M*_w_ = 90 000), *N*,*N*-dimethylformamide (DMF, AR), nickel nitrate [Ni(NO_3_)_2_·6H_2_O, AR] and cobalt acetate tetrahydrate [Co(CH_3_COO)_2_·4H_2_O, AR] were used in the experiments. Distilled water was made in our lab.

### Fabrication of NiO nanowire-in-nanotube structured nanofibers

1.0000 g of Ni(CH_3_COO)_2_·4H_2_O was dissolved in 7.0000 g of DMF, and then 2.0000 g of PVP was added into the above solution under magnetic stirring for 12 h to form uniform green-transparent spinning solution with a certain viscosity. In the electrospinning solution, the mass ratio of Ni(CH_3_COO)_2_·4H_2_O, PVP and DMF was fixed as 10 : 20 : 70. Subsequently, the electrospinning was carried out at ambient temperature by using a conventional single-spinneret electrospinning setup with the positive direct current (DC) voltage of 13 kV and the spinning distance of 18 cm. Then, Ni(CH_3_COO)_2_/PVP composite nanofibers were obtained on the collector through the above process with the volatilization of solvent.

The as-prepared Ni(CH_3_COO)_2_/PVP composite nanofibers were heat-treated from ambient temperature (20 °C) to 200 °C with a heating rate of 1 °C min^−1^ and then remained for 2 h at 200 °C (first-stage calcination, named as pre-oxidation process), after that, the temperature was raised to 450 °C with the same heating rate of 1 °C min^−1^ and remained for 2 h (second-stage calcination, denoted as oxidation process). Thereafter, the temperature was reduced to 200 °C at a cooling rate of 1 °C min^−1^ followed by natural cooling down to room temperature, and thus NiO nanowire-in-nanotube structured nanofibers were successfully obtained.

### Comparative and conditional experiments

In order to obtain the optimum preparation parameters for NiO nanowire-in-nanotube structured nanofibers, the influence of different preparation conditions such as pre-oxidation temperature, pre-oxidation duration time, heating rate, oxidation temperature, oxidation duration time and various inorganic salts on the morphology of the sample were studied, and a series of conditional experiments were conducted and detailedly listed in Table 1.

**Table 1:** Experimental conditions for preparing different samples

Samples	Pre-oxidation temperature (°C)	Pre-oxidation duration time (h)	Heating rate (°C min^−1^)	Oxidation temperature (°C)	Oxidation duration time (h)	Inorganic salts	Morphology
S1	150	2	1	450	2	Ni(CH_3_COO)_2_·4H_2_O	Nanowire-in-nanotube structured nanofibers
S2	200	2	1	450	2	Ni(CH_3_COO)_2_·4H_2_O	Nanowire-in-nanotube structured nanofibers
S3	250	2	1	450	2	Ni(CH_3_COO)_2_·4H_2_O	Nanowire-in-nanotube structured nanofibers
S4	200	1	1	450	2	Ni(CH_3_COO)_2_·4H_2_O	Nanowire-in-nanotube structured nanofibers
S5	200	4	1	450	2	Ni(CH_3_COO)_2_·4H_2_O	Nanowire-in-nanotube structured nanofibers
S6	200	6	1	450	2	Ni(CH_3_COO)_2_·4H_2_O	Nanowire-in-nanotube structured nanofibers
S7	200	2	0.5	450	2	Ni(CH_3_COO)_2_·4H_2_O	Solid nanofibers
S8	200	2	3	450	2	Ni(CH_3_COO)_2_·4H_2_O	Broken nanofibers
S9	200	2	5	450	2	Ni(CH_3_COO)_2_·4H_2_O	Broken nanofibers
S10	—	—	1	150	2	Ni(CH_3_COO)_2_·4H_2_O	Solid nanofibers
S11	—	—	1	200	2	Ni(CH_3_COO)_2_·4H_2_O	Solid nanofibers
S12	—	—	1	250	2	Ni(CH_3_COO)_2_·4H_2_O	Solid nanofibers
S13	—	—	1	450	2	Ni(CH_3_COO)_2_·4H_2_O	Hollow nanofibers
S14	200	2	1	450	2	Ni(NO_3_)_2_·6H_2_O	Nanowire-in-nanotube structured nanofibers
S15	200	2	1	380	2	Co(CH_3_COO)_2_·4H_2_O	Nanowire-in-nanotube structured nanofibers

Samples S1–S3 were obtained at different pre-oxidation temperatures by two-stage calcination of Ni(CH_3_COO)_2_/PVP composite nanofibers. S4–S6 were prepared by changing the pre-oxidation duration time *via* two-stage calcination of Ni(CH_3_COO)_2_/PVP composite nanofibers. Samples S7–S9 were obtained in the same conditions except for the different heating rates. S10–S13 were synthesized at different oxidation temperatures by one-stage calcination of Ni(CH_3_COO)_2_/PVP composite nanofibers without undergoing pre-oxidation. S14 and S15 were fabricated by using different kinds of inorganic salts *via* two-stage calcination of corresponding composite nanofibers.

### Characterization methods

X-ray diffraction (XRD) patterns were collected by using a Rigaku D/max-RA X-ray diffractometer operating at 40 kV and 30 mA with the Cu Kα radiation and Ni filter (*λ* = 0.15418 nm). The scanning electron microscope (FESEM, JSM-7610F) and transmission electron microscope (TEM; JEM-2100 Plus) were used to observe the morphologies and sizes of the samples. The elemental analysis was performed by X-MaxN80 energy dispersive X-ray spectrometer (EDS) attached to SEM. Thermogravimetric and differential scanning calorimetry (TG-DSC) analysis was carried out on a Q600 thermal analyzer in air atmosphere. The specific surface area of products was measured by ASAP 2020 instrument.

### Hydrogen production measurements

Experiments for photocatalytic water splitting into hydrogen were performed in a Labsolar-IIIAG photocatalytic system device (Beijing Bofeilai Technology Co., Ltd) by external visible light irradiation. The light source was a xenon lamp (300 W, PLSSXE300/300UV, China) equipped with a cut off filter L38 (380 < *λ* < 750 nm). Before testing, 0.1 g of the photocatalyst (NiO nanowire-in-nanotube structured nanofibers and NiO hollow nanofibers), 25 mL of methyl alcohol and 75 mL of tap water were successively added into a 200 mL quartz cuvette to ensure uniform dispersion of the sample under vigorous magnetic stirring. Then the suspension was degassed by evacuation. Throughout the experiment, the amount of produced gas was sampled intermittently, and the hydrogen content was measured by gas chromatography (GC7900, Tianmei Techcomp Ltd., thermal conductivity detector, using nitrogen as carrier gas).

## Results and discussion

### Thermal analysis

**Fig. 1:**
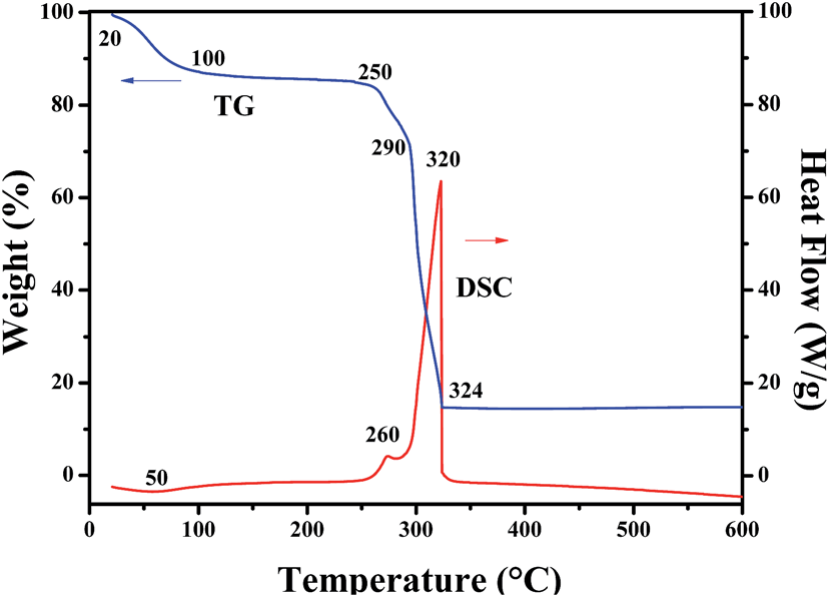
TG and DSC curves of Ni(CH_3_COO)_2_/PVP composite nanofibers.

TG and DSC curves of Ni(CH_3_COO)_2_/PVP composite nanofibers, as seen in Fig. 1. Due to the volatilization of the residual solvent and the surface adsorbed water, the Ni(CH_3_COO)_2_/PVP composite nanofibers lose about 12.55% of their initial weight when the temperature arises from 20 °C to 100 °C accompanied by a wide endothermic peak at 50 °C in the DSC curve. With the continuous rise in temperature to 250 °C, Ni(CH_3_COO)_2_/PVP composite nanofibers slightly lose their weight due to the oxidation of Ni(CH_3_COO)_2_ to form NiO. When the temperature reaches up to 250 °C, Ni(CH_3_COO)_2_ is intensively decomposed to form NiO, gaseous H_2_O and CO_2_, together with obvious weight loss in TG curve and an exothermic peak at 260 °C in DSC curve. Afterwards, PVP begins to decompose at 290 °C, and the decomposition process is completed at 324 °C. Major weight loss and heat release occur in this process. No weight loss in TG curve and thermal peak in DSC curve are detected when temperature is over 324 °C, meaning that stable inorganic oxide can be obtained above 324 °C, and the total weight loss percentage is 85%.

### XRD analysis

**Fig. 2:**
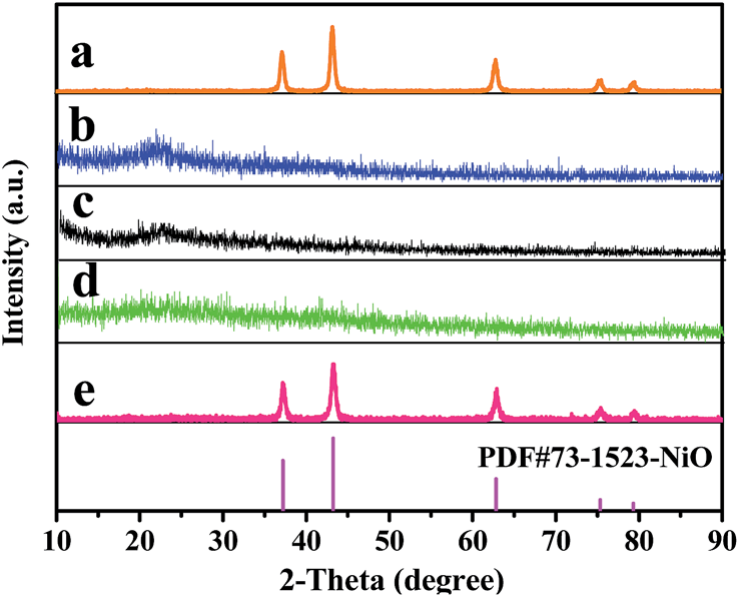
XRD patterns of samples S2 (a), S10 (b), S11 (c), S12 (d) and S13 (e) with PDF standard card of NiO.

Fig. 2 indicates XRD patterns of samples obtained at different preparative conditions. Fig. 2a displays the XRD patterns of S2 prepared by two-stage calcination agree well with the PDF standard diffraction lines (PDF#73-1523) and no impurity peaks are detected, meaning that pure cubic phase NiO with the space group of *Fm3̄m* is obtained.

In order to study the crystalline phases of products undergone the pre-oxidation process, the XRD patterns of samples S10–S12 were also gained, as seen in Fig. 2b–d. One can see that only amorphous peak at *ca.* 22° is found, indicating that no crystalline NiO is formed or the weight percentages of crystalline NiO in these samples do not exceed 5% (limit of XRD detection) at 150 °C to 250 °C.

Furthermore, it can be observed from Fig. 2e that the XRD patterns of S13 prepared by one-stage calcination are consistent with those of PDF standard card of NiO (PDF#73-1523), implying that pure phase NiO is also acquired.

### SEM and TEM analyses

**Fig. 3:**
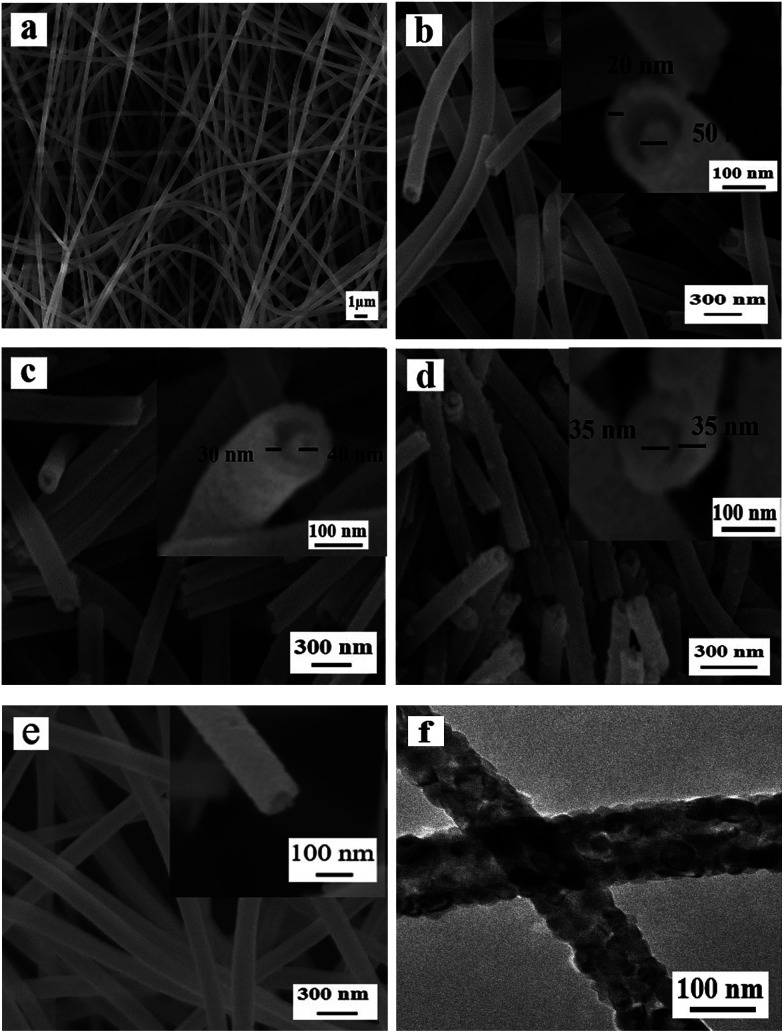
SEM images of Ni(CH_3_COO)_2_/PVP composite nanofibers (a), NiO nanowire-in-nanotube structured nanofibers S1 (b), S2 (c), S3 (d) and NiO hollow nanofibers S13 (e); TEM image of NiO hollow nanofibers S13 (f).

Fig. 3 manifests the morphologies of Ni(CH_3_COO)_2_/PVP composite nanofibers and samples S1–S3 and S13 obtained by different calcination process. One can see that Ni(CH_3_COO)_2_/PVP composite nanofibers have smooth surface and uniform dispersity, as indicated in Fig. 3a. Fig. 3b–d reveals the morphologies of samples S1–S3 obtained at different pre-oxidation temperatures (S1: 150 °C, S2: 200 °C, S3: 250 °C) by two-stage calcination are NiO nanowire-in-nanotube structured nanofibers that consist of a shell of nanotube and a core of nanowire. With the increase of the pre-oxidation temperature, the shell of the NiO nanowire-in-nanotube structured nanofibers is gradually thickened and becomes rough, and the space between the nanowire and the nanotube is gradually reduced, as seen in Fig. 3b–d.

Fig. 3e and f indicate the morphology of S13 prepared by one-stage calcination of Ni(CH_3_COO)_2_/PVP composite nanofibers without pre-oxidization process. It is observed that hollow structured nanofibers, rather than nanowire-in-nanotube structured nanofibers, are obtained. Therefore, it can be concluded that pre-oxidation process plays an important role in the formation of NiO nanowire-in-nanotube structured nanofibers. Furthermore, SEM observation demonstrates that the diameters of Ni(CH_3_COO)_2_/PVP composite nanofibers, S1, S2, S3 and S13 are 293 ± 1.43 nm, 160 ± 3 nm, 130 ± 0.99 nm, 111 ± 1.33 nm and 120 ± 3.17 nm, respectively. It is found that with the increase of the pre-oxidation temperature, the diameters of the samples are gradually decreased. To investigate the influence of other conditions on the morphology of the products, we choose 200 °C as the optimal pre-oxidation temperature in the subsequent work.

**Fig. 4:**
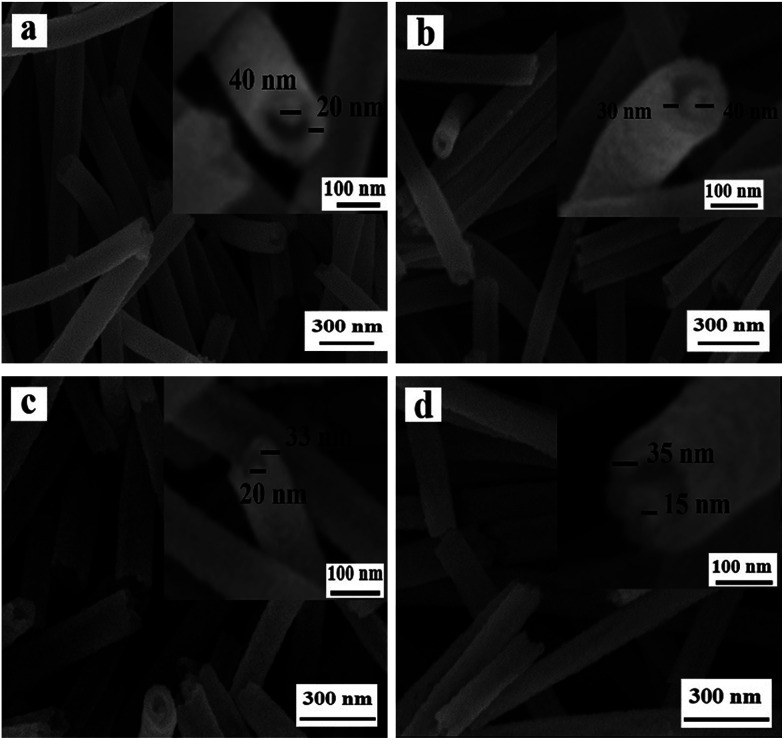
SEM images of NiO nanowire-in-nanotube structured nanofibers S4 (a), S2 (b), S5 (c) and S6 (d).

Fig. 4 reveals the SEM images of NiO nanowire-in-nanotube structured nanofibers obtained at different pre-oxidation duration time of 1 h, 2 h, 4 h, 6 h (S4, S2, S5, S6) by two-stage calcination of Ni(CH_3_COO)_2_/PVP composite nanofibers. It is easy to find that all these samples are NiO nanowire-in-nanotube structured nanofibers. With the increase of pre-oxidation duration time, the surface of nanowire-in-nanotube structured nanofibers gradually becomes rough. It is also found that the diameters of S4, S5 and S6 respectively are 138 ± 3.36 nm, 98 ± 0.79 nm and 95 ± 0.35 nm, indicating that the diameters of the samples are gradually reduced with the increase of the pre-oxidation duration time. Thus, 2 h is selected as the optimum pre-oxidation duration time in the following study.

**Fig. 5:**
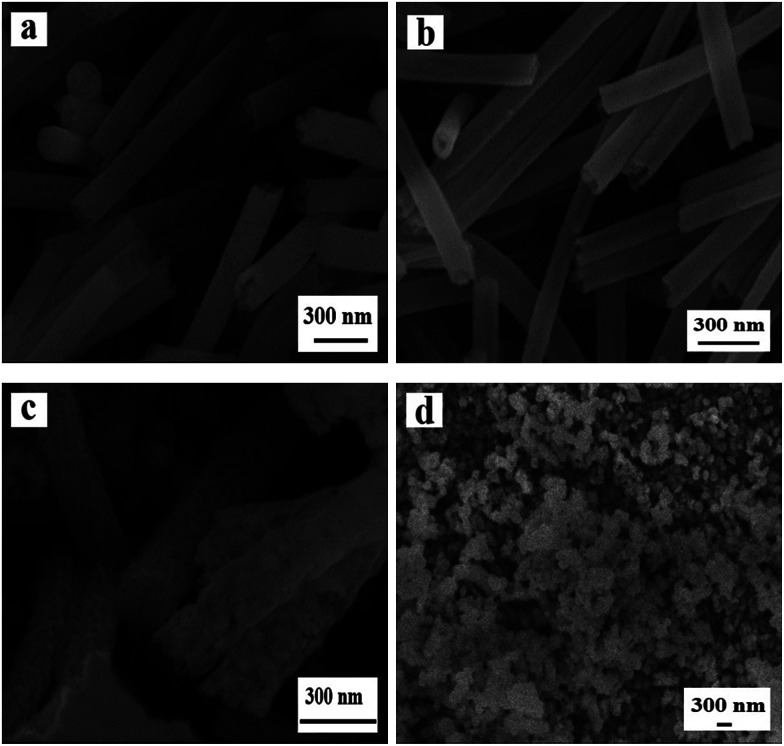
SEM images of samples S7 (a), S2 (b), S8 (c) and S9 (d).

The SEM images of samples S7, S2, S8, S9 fabricated at different heating rate (0.5 °C min^−1^, 1 °C min^−1^, 3 °C min^−1^, 5 °C min^−1^) by two-stage calcination of Ni(CH_3_COO)_2_/PVP composite nanofibers, are displayed in Fig. 5. When the heating rate is 0.5 °C min^−1^, NiO solid nanofibers, rather than nanowire-in-nanotube structured nanofibers, are acquired. Fig. 5b demonstrates the appropriate heating rate (1 °C min^−1^) is beneficial to form nanowire-in-nanotube structure. However, over high heating rate (3 °C min^−1^, 5 °C min^−1^) will lead to the structural damage of the products, as indicated in Fig. 5c and d. The above analyses indicate that the heating rate has a great impact on the formation of NiO nanowire-in-nanotube structured nanofibers. Thus, the heating rate of 1 °C min^−1^ is the best condition for the preparation of NiO nanowire-in-nanotube structured nanofibers.

**Fig. 6:**
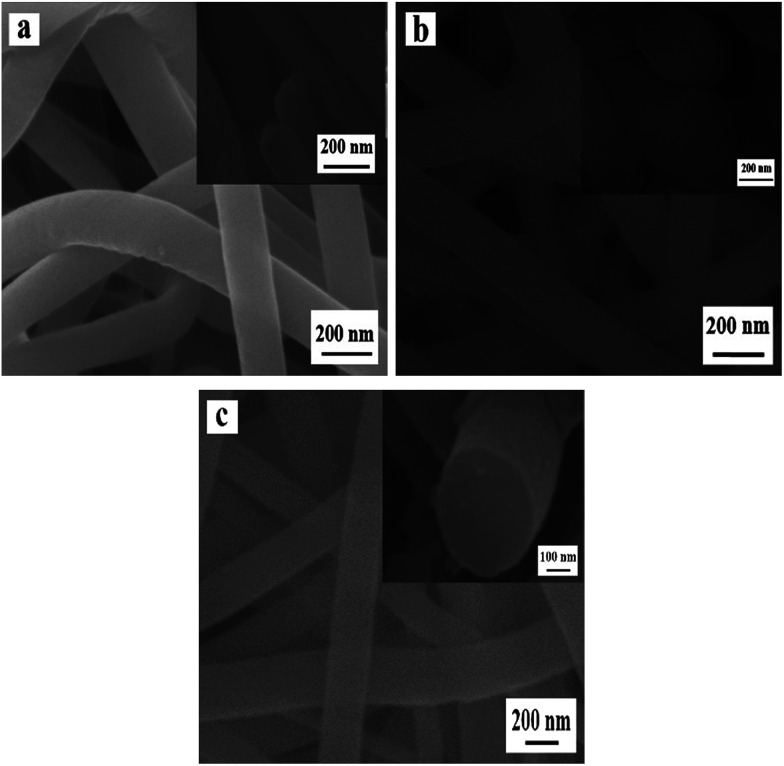
SEM images of samples S10 (a), S11 (b) and S12 (c).

Fig. 6 shows the SEM images of samples obtained by oxidizing Ni(CH_3_COO)_2_/PVP composite nanofibers at 150 °C (a), 200 °C (b) and 250 °C (c) for 2 h. The samples are solid nanofibers regardless of the oxidation temperature of 150 °C, 200 °C and 250 °C, indicating that the PVP in original composite nanofibers does not decomposed and the nanowire-in-nanotube structure is unformed at the ranges of calcination temperatures. Nevertheless, the diameters of these samples are slightly decreased with the increased calcination temperature, which are measured to be 142 ± 4.7 nm (S10), 127 ± 2.22 nm (S11) and 125 ± 1.8 nm (S12), respectively. This is maybe because the Ni(CH_3_COO)_2_ on the surface of the nanofibers is decomposed to NiO, gaseous H_2_O and CO_2_, which causes slight decrease in diameter, whereas the Ni(CH_3_COO)_2_ in the inner nanofibers is barely decomposed due to the non-contact with abundant oxygen.

**Fig. 7:**
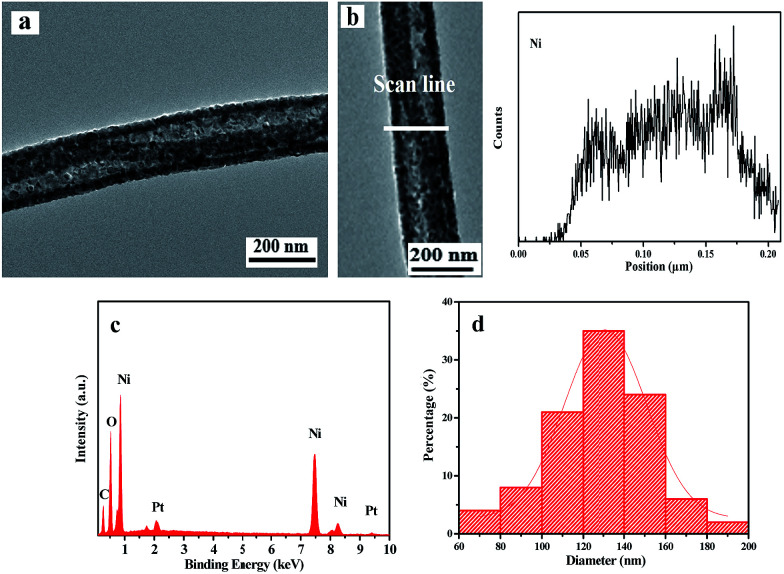
TEM image (a) and EDS line-scan analysis (b) of a single NiO nanowire-in-nanotube structured nanofiber; EDS spectrum (c) and histogram of diameters distribution (d) of NiO nanowire-in-nanotube structured nanofibers.

Based on the above experimental results, it can be concluded that the optimum preparation conditions for NiO nanowire-in-nanotube structured nanofibers are as following: 200 °C for 2 h with a heating rate of 1 °C min^−1^ for the pre-oxidation process, and 450 °C for 2 h with the same heating rate of 1 °C min^−1^ for oxidation process. Fig. 7 displays the TEM image, EDS line-scan analysis, EDS spectrum, and histogram of diameters distribution of NiO nanowire-in-nanotube structured nanofibers obtained under the optimum preparation conditions. As illustrated in Fig. 7a, the diameters of nanowire and nanotube in the NiO nanowire-in-nanotube structured nanofibers are about 40 and 130 nm, respectively. In order to further prove the nanowire-in-nanotube structure and compositions, TEM-EDS line scan analysis was carried out, where Ni element represents NiO, as presented in Fig. 7b. It is found that elemental Ni locates in the whole nanowire-in-nanotube structured nanofiber, and the two edges of the nanotube and the nanowire have larger amount of Ni than the space between the nanowire and nanotube, which is consistent with the structure of nanowire-in-nanotube structured nanofibers. Furthermore, Fig. 7c depicts O and Ni are the main elements in NiO nanowire-in-nanotube structured nanofibers. The average outer diameter of NiO nanowire-in-nanotube structured nanofibers is 130 ± 0.99 nm (Fig. 7d).

**Fig. 8:**
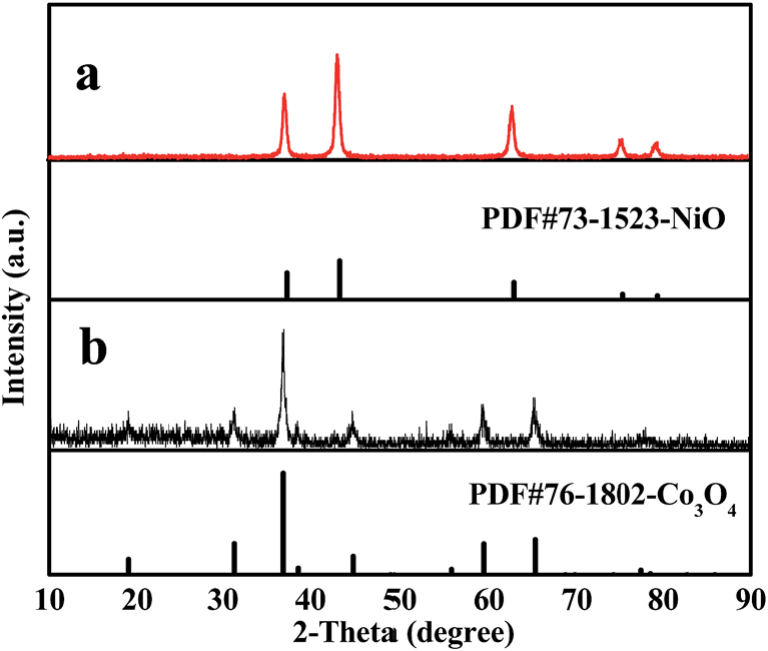
XRD patterns of samples S14 (a) and S15 (b) with PDF standard cards of NiO and Co_3_O_4_.

**Fig. 9:**
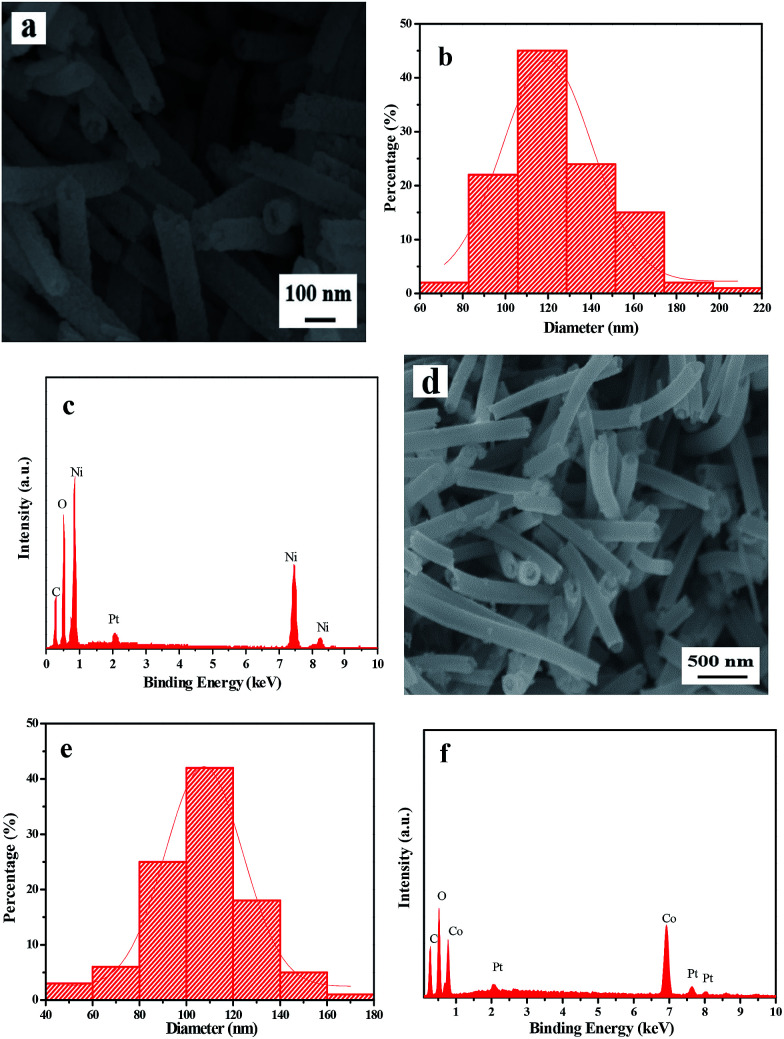
SEM images (a and d), diameters distribution histograms (b and e) and EDS spectra (c and f) of NiO (S14, a–c) and Co_3_O_4_ (S15, d–f) nanowire-in-nanotube structured nanofibers.

In order to demonstrate the universality of this fabrication method, Ni(CH_3_COO)_2_·4H_2_O is respectively replaced by Ni(NO_3_)_2_·6H_2_O and Co(CH_3_COO)_2_·4H_2_O, using the identical optimum pre-oxidation and oxidation conditions. Fig. 8a and b respectively demonstrate the XRD patterns of pure phase NiO and Co_3_O_4_ nanostructures fabricated by two-stage calcination of Ni(NO_3_)_2_/PVP and Co(CH_3_COO)_2_/PVP composite nanofibers. It can be obviously found that NiO and Co_3_O_4_ nanowire-in-nanotube structured nanofibers are obtained, as seen in Fig. 9a and d. Fig. 9c shows that O and Ni are the main elements in NiO nanowire-in-nanotube structured nanofibers. The presence of Co and O corresponds to Co_3_O_4_ nanowire-in-nanotube structured nanofibers, as seen in Fig. 9f. The diameters of NiO and Co_3_O_4_ nanowire-in-nanotube structured nanofibers are 120 ± 3.17 nm and 107 ± 0.56 nm, respectively, as shown in Fig. 9b and e. The above analyses demonstrate that this technique is of certain universality for preparing inorganic metallic oxide nanowire-in-nanotube structured nanofibers.

### Possible formation mechanism of NiO nanowire-in-nanotube structured nanofibers

**Fig. 10:**
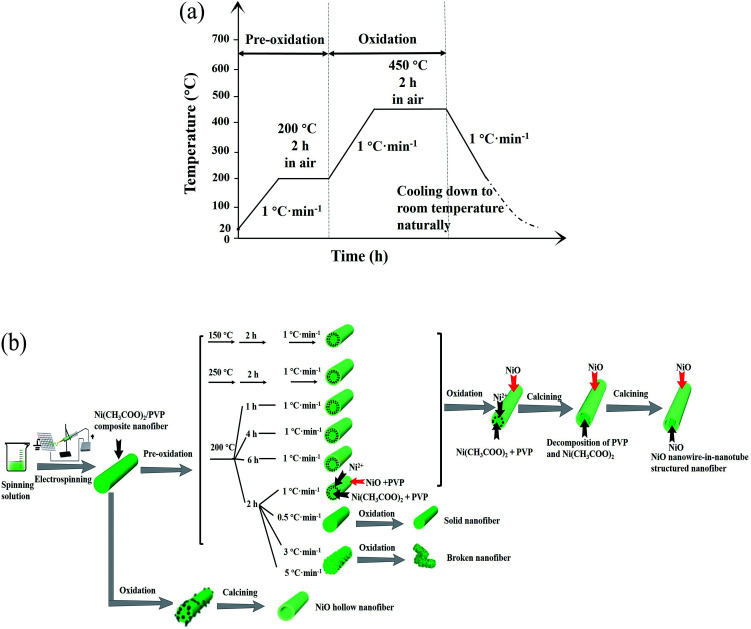
Schematic diagrams of heat-treatment procedure (a) and formation mechanism (b) for NiO nanowire-in-nanotube structured nanofiber.

Fig. 10a shows the flow diagram of heat-treatment procedure for preparing NiO nanowire-in-nanotube structured nanofibers. According to the above results, possible formation mechanism of the NiO nanowire-in-nanotube structured nanofibers is proposed, as indicated in Fig. 10b. Firstly, Ni(CH_3_COO)_2_/PVP composite nanofibers are formed by the traditional electrospinning process using the spinning solution containing PVP, DMF and Ni(CH_3_COO)_2_. At the moment, the composite nanofiber is a solid nanofiber, and PVP plays the role of fiber framework. Secondly, Ni(CH_3_COO)_2_/PVP composite nanofibers are calcined at 200 °C for 2 h (namely pre-oxidation process), and then the temperature is raised to 450 °C and keeps for 2 h (namely oxidation process). In the pre-oxidation process, Ni(CH_3_COO)_2_ on the surfaces of Ni(CH_3_COO)_2_/PVP composite nanofibers first begins to gradually decompose and oxidize to NiO rather than that in the inside of the composite nanofibers because the Ni(CH_3_COO)_2_ on the fiber surfaces could directly contact with oxygen. As a consequence, a NiO shell together with PVP is formed on the surface of each nanofiber. As the pre-oxidation time goes by, the Ni^2+^ ions near the surface of composite nanofiber are gradually attracted to the NiO shell so that the NiO crystal can grow up. Thus, the concentration of Ni^2+^ near the surface of composite nanofiber becomes lower and lower, and the NiO shell turns to denser and denser. Afterwards, in the oxidation process, PVP begins to decompose at 290 °C and produce CO_2_ and H_2_O, accompanied by the decomposition and oxidization of the residual Ni(CH_3_COO)_2_ to NiO. In this process, pre-generated NiO takes place the role of PVP as fiber framework. Moreover, a space between the surface and core of each nanofiber is formed because almost all of the Ni^2+^ ions near the surface of composite nanofiber move to the fiber surface, leaving only PVP which is eliminated by oxidation process. On the other hand, the Ni^2+^ ions in the core of each nanofiber are oxidized to form a NiO nanowire in the outer NiO nanotube. Based on the above formation mechanism of NiO nanowire-in-nanotube structured nanofibers, it is rational that the thickness of the nanotube of each NiO nanowire-in-nanotube structured nanofiber is increased with raising pre-oxidation temperature or extending pre-oxidation duration time, as seen in Fig. 3 and 4, because more Ni^2+^ ions can move to the surface of the composite nanofiber.

It has been found that heating rate also strongly affect the morphology of the products. When the heating rate is 0.5 °C min^−1^, NiO solid nanofibers are formed. The reason is that the inside and surface of the composite nanofiber can fully contact with oxygen to form NiO because there has been sufficient reaction time before the temperature rises to the decomposition temperature of PVP. By contrast, over high heating rate (3 °C min^−1^, 5 °C min^−1^) causes destruction of the morphology of the nanofibers. That is because when the heating rate is too high, PVP and Ni(CH_3_COO)_2_ decompose so fast that lots of gases are rapidly produced, which impedes nanoparticles from mutually connect to form nanofibers.

It has been also discovered that NiO hollow nanofibers are formed when pre-oxidation process is not carried out. The possible formation mechanism of NiO hollow nanofibers is as following: in the process of heating, lots of voids appear in the surface of the composite nanofibers due to the volatilization of residual DMF in the nanofibers. With the increase of temperature, Ni(CH_3_COO)_2_ on the surface of each nanofiber begins to decompose, and thus a porous NiO shell are generated on the surface of nanofiber. Before the NiO shell becomes dense, PVP starts to decompose due to the absence of pre-oxidation process, which causes the Ni^2+^ ions inside the nanofiber are transported to the fiber surface by the gases generated from the PVP.^26^ Finally, Ni^2+^ ions are enriched in the shell of the nanofiber, and thus NiO hollow nanofibers are obtained after calcination.

### Photocatalytic activity and mechanism

**Fig. 11:**
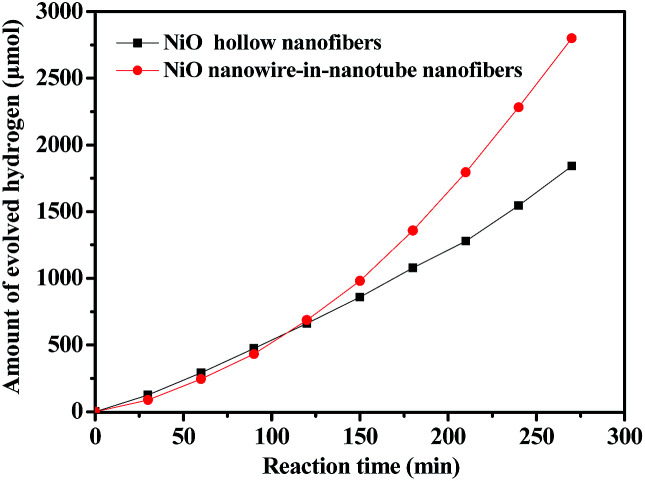
Photocatalytic water splitting activities of NiO nanowire-in-nanotube structured nanofibers (S2) and NiO hollow nanofibers (S13).

**Fig. 12:**
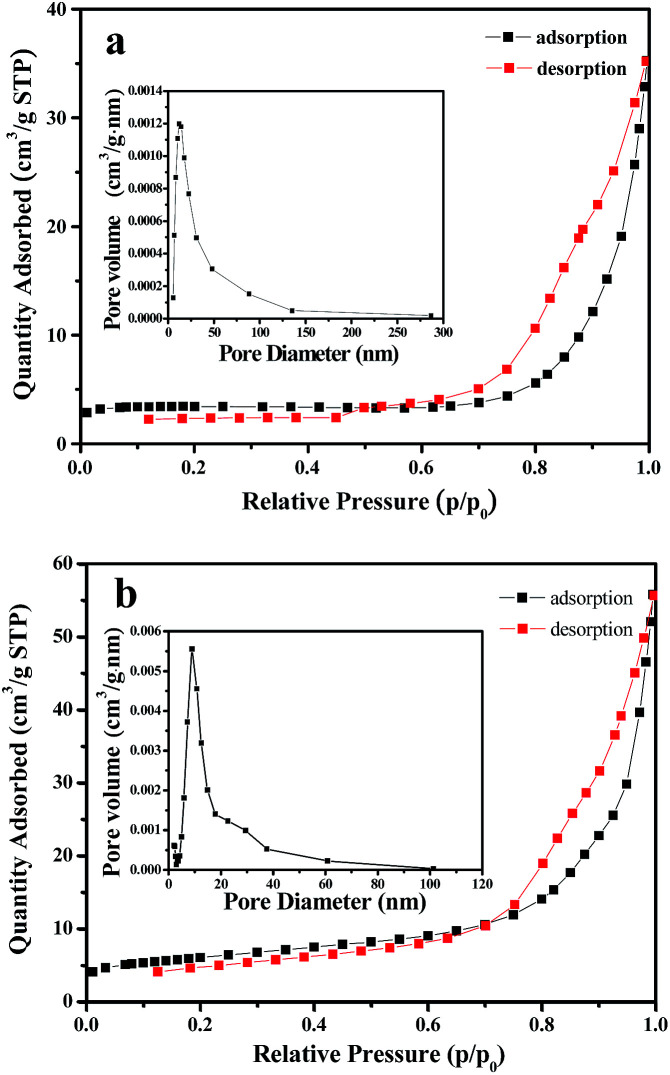
Nitrogen adsorption–desorption isotherm and pore diameter distribution of NiO nanowire-in-nanotube structured nanofibers (a) and NiO hollow nanofibers (b).

Fig. 11 reflects a comparison of the hydrogen production activity from water splitting by using NiO nanowire-in-nanotube structured nanofibers and NiO hollow nanofibers under visible light illumination (*λ* > 380 nm) with methanol as a sacrifice. It can be seen that the hydrogen production rate of NiO nanowire-in-nanotube structured nanofibers and NiO hollow nanofibers is respectively 622 μmol h^−1^ and 472 μmol h^−1^ under the same mass. The amount of hydrogen produced by NiO nanowire-in-nanotube structured nanofibers is 1.32 times higher than that of NiO hollow nanofibers. Fig. 12 demonstrates nitrogen adsorption–desorption isotherm and pore diameter distribution of NiO nanowire-in-nanotube structured nanofibers and NiO hollow nanofibers. The specific surface area and pore diameter of NiO nanowire-in-nanotube structured nanofibers (20.81 m^2^ g^−1^, 24 nm) are bigger than those of NiO hollow nanofibers (9.95 m^2^ g^−1^, 12 nm). It has been known that the H_2_-production activity from water splitting strongly depends on the microstructure of NiO.^27,28^Fig. 14 displays the total surface area of the NiO nanowire-in-nanotube structured nanofibers includes the surface area of the embedded nanowires and the inner and outer surface areas of the nanotubes, leading to the fact that NiO nanowire-in-nanotube structured nanofibers have bigger surface area than NiO hollow nanofibers, which is confirmed by the above specific surface area data. Hence, NiO nanowire-in-nanotube structured nanofibers can absorb more light and adsorb more water and methanol molecules than NiO hollow nanofibers, resulting in the fact that hydrogen production rate of NiO nanowire-in-nanotube structured nanofibers is faster than that of NiO hollow nanofibers. On the other hand, bigger pore diameter is beneficial to enhance the absorption efficiency of light and accelerate the flow rate of the water molecules,^29^ which also causes higher hydrogen production rate of NiO nanowire-in-nanotube structured nanofibers. Nonetheless, at the beginning of the reaction, the hydrogen production rate of the NiO hollow nanofibers is higher than that of NiO nanowire-in-nanotube structured nanofibers. This may be because nanowires in the NiO nanowire-in-nanotube structured nanofibers block water molecules and methanol molecules from getting into the NiO nanowire-in-nanotube structured nanofibers, and thus the inner surfaces of the NiO nanowire-in-nanotube structured nanofibers are rarely used. As the reaction time increases, water molecules and methanol molecules gradually enter into the NiO nanowire-in-nanotube structured nanofibers, the large inner surfaces greatly promote the reaction. Therefore, the H_2_-production activity is accelerated.

**Fig. 13:**
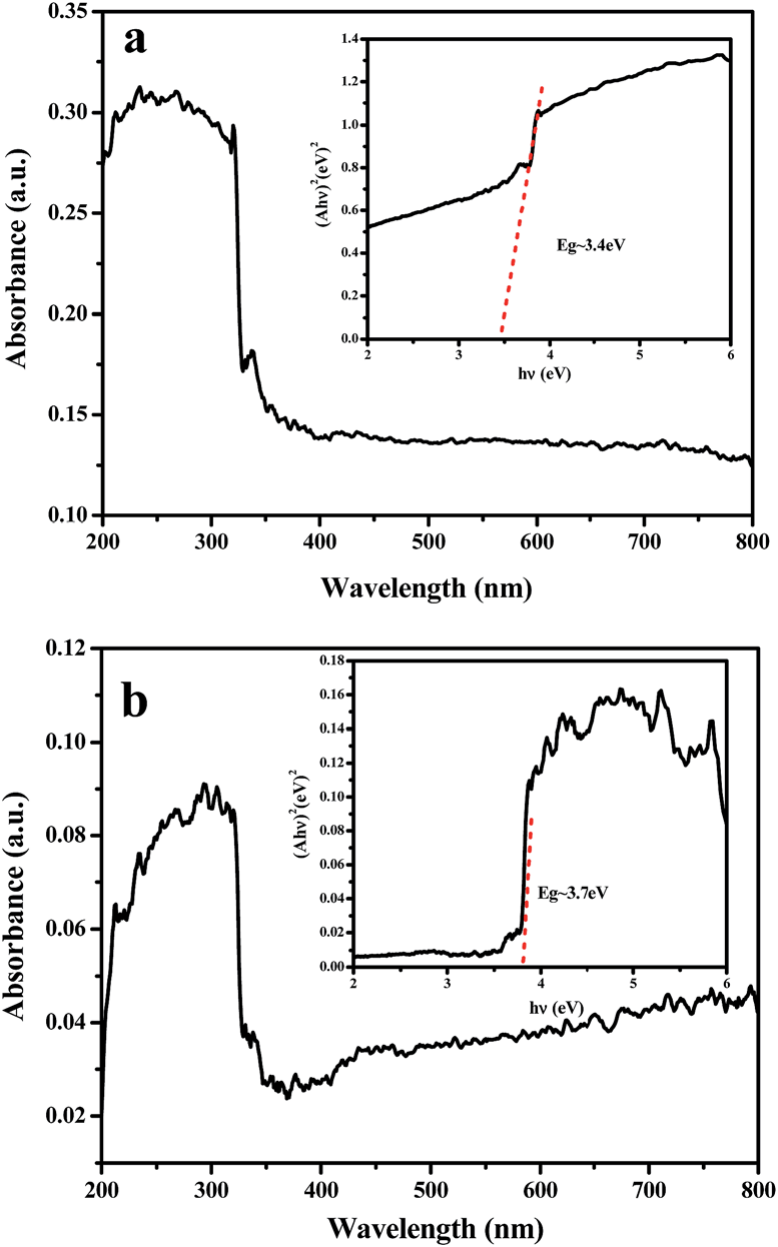
UV-vis absorption spectra of NiO nanowire-in-nanotube structured nanofibers (a) and NiO hollow nanofibers (b). The inset is the plot of (*Ahν*)^2^*versus hν*.

**Fig. 14:**
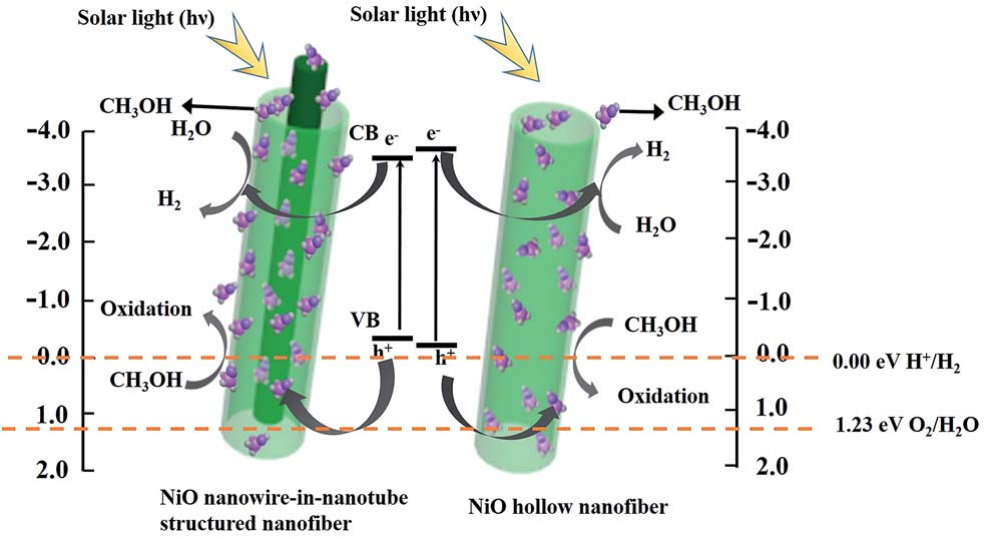
Possible mechanism for photocatalytic H_2_ evolution over NiO nanowire-in-nanotube structured nanofibers (S2) and NiO hollow nanofibers (S13) under visible light irradiation.

Fig. 13 shows the UV-vis spectroscopy of the NiO nanowire-in-nanotube structured nanofibers and NiO hollow nanofibers. It can be seen that NiO nanowire-in-nanotube structured nanofibers exhibit stronger absorption in the range of visible light than NiO hollow nanofibers, which is due to the larger specific area of NiO nanowire-in-nanotube structured nanofibers. Generally, larger specific area provides more highly active sites for H_2_ evolution, which facilitates photocatalytic reaction. For crystalline semiconductors, the band gap energies of the samples can be estimated from a plot of (*αhν*)^2^*versus* photon energy (*hν*). The indirect band gap energies of the samples are similar to the intercept of the tangent to the plot, and the band gap of the sample can be calculated by the formula:^22^(*αhν*)^2^ = *B*(*hν* − *E*_g_)In which *B* is the absorption constant for indirect transitions, absorbance (*A*) is proportional to the absorption coefficient (*α*). Here, *α* is replaced by *A*. The insets of Fig. 13 display that the band gaps of NiO nanowire-in-nanotube structured nanofibers and NiO hollow nanofibers are 3.4 and 3.7 eV, respectively, which are close to the reported values of NiO (3.6–4.0 eV).^22^

The possible mechanism for photocatalytic hydrogen generation over NiO nanostructures is as following: the CB position of NiO can be expressed empirically by the formula:^30^*E*_CB_ = *X* − *E*^C^ − 1/2*E*_g_where *E*_g_ is the band gap energy of the NiO, E^C^ is the energy of free electrons on the hydrogen scale (*ca.* 4.5 eV), *X* is the electronegativity of NiO.^31^ CB potential of NiO nanowire-in-nanotube structured nanofibers and NiO hollow nanofibers are calculated to be −3.6 eV and −3.79 eV, respectively. The VB potential of NiO can be calculated by the formula:*E*_VB_ = *E*_CB_ + *E*_g_.

After calculation, the VB potential of NiO nanowire-in-nanotube structured nanofibers and NiO hollow nanofibers are −0.2 eV and −0.09 eV, respectively, as indicated in Fig. 14. The band gap of NiO nanowire-in-nanotube structured nanofibers (3.4 eV) is much smaller than that of NiO hollow nanofibers (3.7 eV). The narrower the band gap of the sample, the easier the electrons are excited in the valence band, which results in the fact that the photocatalytic reaction on NiO nanowire-in-nanotube structured nanofibers is more easily to occur than that on NiO hollow nanofibers under the same energy of light. Under visible light irradiation, methanol, which acts as a sacrificial electron donor, can fast remove the photo-generated holes and/or photo-generated oxygen in an irreversible fashion, thereby restraining electron–hole recombination and/or the reverse reaction of H_2_ and O_2_.^32^ When NiO nanofibers are exposed to visible light, the energy of a photon is absorbed by an electron in the valence band of NiO nanofibers. The photogenerated electron (e^−^) is excited to the conduction band and simultaneously leaves behind a positive hole (h^+^) in the valence band. Subsequently, OH^−^ and H_2_ are produced to reduce a water molecules by a photogenerated electron (e^−^). At the same time, the separation efficiency of the electron–hole pairs is enhanced, due to the reaction of the CH_3_OH reagents with the photogenerated hole (h^+^). It was proposed that the reaction equation for production-H_2_ from water splitting is as follows:^33^

1 NiO + *hν* → h^+^ + e^−^

2 h^+^ + H_2_O → ˙OH + H^+^

3 CH_3_OH + ˙OH → ˙CH_2_OH + H_2_O

4 ˙CH_2_OH → HCHO + H^+^ + e^−^

5 2H_2_O + 2e^−^ →H_2_ + 2OH^−^

## Conclusions

We propose a simple and universal technique to synthesize metallic oxide nanowire-in-nanotube structured nanofibers by two-stage calcination of electrospinning-made composite nanofibers. NiO nanowire-in-nanotube structured nanofibers with space group of *Fm3̄m* were fabricated by this fabrication method for the first time. The outer diameter and wall thickness of nanotubes and embedded nanowire diameter are 130 ± 0.99 nm, 30 nm and 40 nm, respectively. NiO nanowire-in-nanotube structured nanofibers used as photocatalyst for water splitting exhibit higher H_2_ evolution rate of 622 μmol h^−1^ than NiO hollow nanofibers of 472 μmol h^−1^ under visible light illumination owing to its special nanostructure. This technique we newly designed and proposed possesses universality and guiding significance to fabricate other nanowire-in-nanotube structured materials. The step for removing middle layer of the as-prepared fibers in conventional methods can be omitted by using our method, so that the preparation procedure is simplified. Moreover, for nanowire-in-nanotube structured materials, the spatial characteristics between embedded nanowire and nanotube may be considered as potential applications in the fields of photocatalysis, drug loading and delivery, sensor, and Li-ion battery.

## Conflicts of interest

There are no conflicts of interest to declare.

## Supplementary Material
